# A Convolution-Based Coding Metasurface for Wide-Angle Beam Steering for Enhanced 5G Wireless Communications

**DOI:** 10.3390/ma18091913

**Published:** 2025-04-23

**Authors:** Jing Wang, Yan Chen, Benxian Wang, Xin Liu, Junfei Gao, Qi Xue, Xiaojun Huang

**Affiliations:** 1 School of Physics and Electronic Engineering, Xinjiang Normal University, Urumqi 830054, China; chenyan_0620@163.com (Y.C.); wbxwangbenxian587@163.com (B.W.);; 2 College of Communication and Information Engineering, Xi’an University of Science and Technology, Xi’an 710054, China; gjf1031@126.com (J.G.); xueqi11032025@163.com (Q.X.)

**Keywords:** coding metasurface, beam steering, convolution operation

## Abstract

With the rapid development of 5G communication technology, there is an increasing demand for high-performance antennas and beam control technologies, making the development of novel metamaterial structures capable of precise electromagnetic wave manipulation a current research hotspot. This paper presents a coding metasurface specifically designed for 5G communication applications, operating at a frequency of 3.5 GHz. The design employs a unique annular metasurface unit structure capable of achieving both single-beam and dual-beam functionalities. Through convolution operations, precise control over the reflection angle is achieved, with an adjustable range from 51.5° to 17.5° and a resolution of 10°. This design overcomes the inherent limitations of traditional gradient coding methods, providing a comprehensive framework for wide-angle reflection control in metasurface design. The research results demonstrate that the coding metasurface can effectively control the reflection direction of electromagnetic waves at 3.5 GHz, exhibiting dual-polarization modulation capabilities and maintaining stable performance under oblique incidence conditions within 20°. Experimental validation confirms the beam control functionality of the design in real-world environments, highlighting its potential to enhance signal reception sensitivity and transmission efficiency in 5G wireless communications. This work opens new avenues for research in reconfigurable and intelligent metasurfaces, with potential applications extending beyond 5G to future 6G networks and Internet of Things (IoT) systems.

## 1. Introduction

Over the past decades, metasurfaces have emerged as a transformative technology in electromagnetic wave manipulation, attracting significant attention from researchers due to their unique electromagnetic properties that are unattainable in natural materials. As two-dimensional planar structures composed of subwavelength-scale artificial units, metasurfaces have demonstrated remarkable capabilities in controlling the propagation direction and phase distribution of electromagnetic waves, thereby enhancing signal strength and extending coverage range in communication systems [1,2,3,4,5,6]. The field witnessed a paradigm shift in 2014 with the introduction of digital coding and programmable metasurfaces, enabling unprecedented digital control over electromagnetic waves [7]. The fundamental 1-bit coding metasurface, comprising two distinct elements marked as ‘0’ and ‘1’ with phase states of 0° and 180°, respectively, has been extended to multi-bit configurations through phase discretization within 360°. This advancement allows for sophisticated directional control of single-beam, dual-beam, and multi-beam patterns [8,9,10]. Furthermore, the integration of digital convolution operations with coding metasurfaces has enabled precise scattering pattern shifts, facilitating beam rotation to desired directions with minimal deformation and enabling arbitrary angle beam scanning in free space [11].

The advent of 5G wireless communication technology, characterized by its high-speed data transmission, low latency, and large network capacity, has revolutionized modern communication paradigms. Within this context, the 3.5 GHz frequency band has emerged as a cornerstone for initial 5G network deployment. Compared to higher frequency bands, such as millimeter wave, the 3.5 GHz band offers distinct advantages including lower propagation losses, extended transmission distances, broader base station coverage, and reduced network deployment costs. Recognizing these benefits, this study focuses on developing a coding metasurface specifically designed for operation at 3.5 GHz, ensuring optimal compatibility with practical 5G deployment scenarios.

Recent advancements in 3.5 GHz metasurface-assisted communication systems have primarily focused on electromagnetic beam control, leveraging the ability of metasurfaces to manipulate phase, amplitude, and polarization characteristics through precise unit structure design [12,13,14,15]. A significant breakthrough occurred in 2023 with the development of a passive lossless metasurface capable of arbitrary multi-beam direction and power control through surface wave manipulation, demonstrating precise control over reflected beams at 30°and 60° with both equal and unequal power distribution [16]. The integration of digital convolution operations has further enhanced metasurface capabilities, enabling flexible control over electromagnetic wave refraction and reflection. This capability proves particularly valuable in addressing coverage blind spots caused by obstacles or propagation losses, effectively extending communication signal coverage and modifying propagation paths. Notable contributions include Zhang et al.’s [17] 2-bit reconfigurable intelligent metasurface-assisted communication system at 3.5 GHz, which achieved multi-beam reflection at various angles with a 40 MHz bandwidth, and Zhao et al.’s [18] innovative three-layer C-type coding metasurface in the terahertz range, demonstrating a 52° deflection angle at 0.71 THz with 76% deflection efficiency through convolution calculations. These advancements collectively underscore the transformative potential of coding metasurfaces in next-generation communication systems.

In this study, we propose an innovative coding metasurface tailored for 5G communication systems, operating at the 3.5 GHz frequency band, which is widely recognized as a key frequency for 5G deployment due to its optimal balance between coverage and data transmission capabilities. The design features a unique annular metasurface unit configuration capable of supporting both single-beam and dual-beam functionalities, providing enhanced flexibility for diverse communication scenarios. The coding sequences of two or three arrays are optimized by using convolution operation, achieving wide-angle beam deflection from 51.5° to 17.5° at intervals of 10°. This significantly expands the angular range of beam control and improves the signal coverage capability. Compared to the traditional design methods that rely on complex optimization algorithms, the design method based on convolution operation proposed in this paper has higher computational efficiency and operability, providing a new idea for the design of metasurfaces.

## 2. Conception and Design

**Figure 1:**
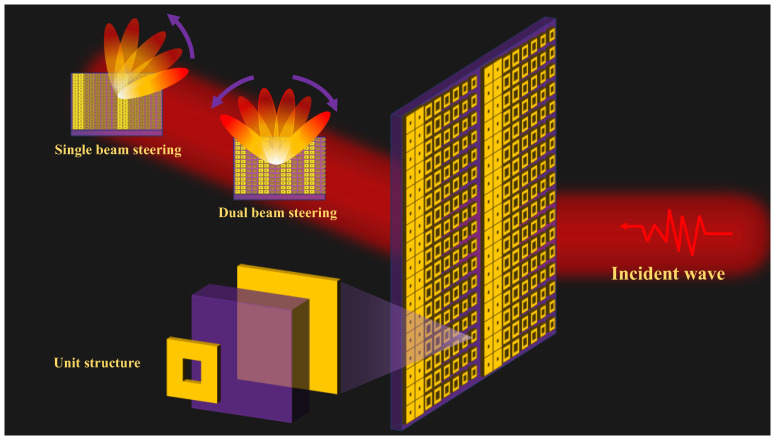
The implementation of functionalities in coding metasurfaces subjected to vertically incident plane waves.

Figure 1 illustrates the functional realization of coding metasurfaces under vertically incident plane waves, demonstrating both single-beam and multi-beam deflection capabilities. By strategically designing the coding metasurface, precise control over beam deflection directions can be achieved, enabling large-angle and flexible beam steering for both single and multiple beams. The image includes a detailed schematic of the metasurface unit structure, highlighting the specific design features that facilitate these advanced functionalities. This schematic provides insight into the structural configuration and operational principles underlying the metasurface’s ability to manipulate electromagnetic waves with high precision.

**Figure 2:**
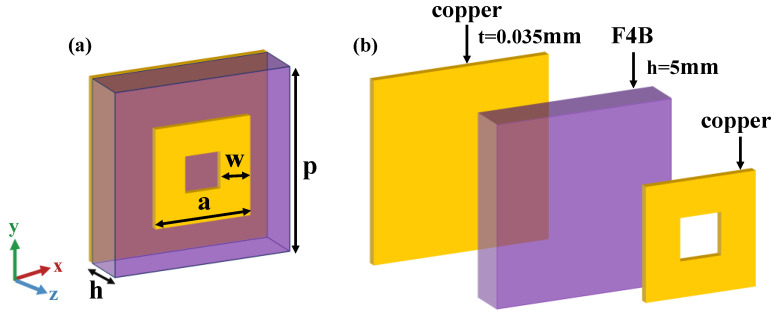
(**a**) Schematic diagram of the metasurface unit structure; (**b**) Cross-sectional view of the metasurface unit.

The coding element is composed of two copper layers, each with a thickness of 0.035 mm, separated by F4B substrates (ε_r_ = 2.2, tan δ = 0.001) with a thickness of h = 5 mm. The top layer features a circular metal patch with an outer side length denoted as ‘a’ and a width of ‘w’. The periodicity of the coding element is set at p = 21.5 mm, as depicted in Figure 2. This annular metasurface structure, initially proposed in [19], serves as the foundation for the current design. In this work, we significantly expand the tunable range of the reflection angle and fabricate a metasurface sample to experimentally validate its performance, thereby advancing the practical application of this innovative design.

The metasurface units are encoded by selecting two distinct units with a phase difference of 180°, characterized by patch side lengths ‘a’ of 21.5 mm and 17.5 mm, respectively. These units are digitally represented as “0” and “1”, forming the basis of a 1-bit coding metasurface. To achieve higher phase resolution, four units with a phase difference of 90° are incorporated, with patch side lengths ‘a’ of 21.5 mm, 18.8 mm, 17.5 mm, and 15.3 mm, corresponding to phase states of 0°, 90°, 180°, and 271°, respectively. These four units are digitally encoded as 0, 1, 2, and 3 (or equivalently represented in binary as “00”, “01”, “10”, and “11”), as comprehensively outlined in Table 1. This multi-bit encoding approach enhances the metasurface’s capability for precise phase manipulation and advanced beam control.

**Figure 3:**
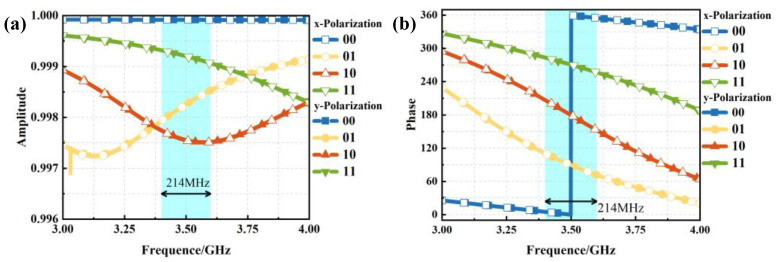
Amplitude and phase response of reflected waves when x- and y-polarized waves are incident along the z-axis (**a**) reflection amplitude; (**b**) reflection phase.

Electromagnetic simulation analysis was performed on the unit structure, and the resulting reflection amplitude and phase characteristics are presented in Figure 3. At the target frequency of 3.5 GHz, x- and y-polarized incident waves were vertically incident along the z-axis. As shown in Figure 3a, the reflection amplitude exceeds 0.97, indicating high reflectivity efficiency. Figure 3b reveals that the reflection phase achieves a 90° phase difference across the four coding states, with a phase deviation maintained within 90° ± 10° over the frequency range of 3.4 to 3.6 GHz, corresponding to an operational bandwidth of 214 MHz. Owing to the centrosymmetric configuration of the unit structure, identical amplitude and phase responses are observed for both x- and y-polarized waves, demonstrating the dual-polarization modulation capability of the designed metasurface unit.

**Figure 4:**
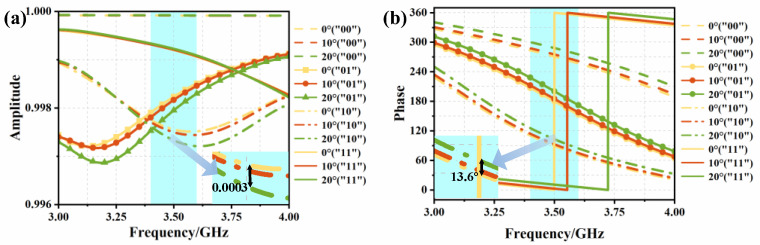
(**a**) Reflection amplitudes of the four states “00”, “01”, “10”, and “11” under different incident angles; (**b**) reflection phases of the four states “00”, “01”, “10”, and “11” under different incident angles.

In practical deployment scenarios, where metasurfaces are employed to enhance signal strength, the transmitter and receiver are typically positioned at different azimuths, resulting in varying incident angles, as signals impinge on the metasurface. This practical consideration necessitates the careful structural design of the metasurface. To address this, we systematically investigated the effects of oblique incidence on the metasurface unit’s performance. Simulations were conducted for incident angles of θ = 0°, 10°, and 20°, with the corresponding reflection characteristics illustrated in Figure 4. The results demonstrate that, for incident angles within 20°, the reflection amplitude exhibits minimal variation (ΔA = 0.0003), while the reflection phase shows a moderate change of 13.6°. These findings indicate that the metasurface unit maintains stable performance under oblique incidence conditions up to 20°, with negligible impact on both reflection amplitude and phase characteristics. This robust angular stability is crucial for practical applications in real-world communication scenarios.

## 3. Results and Discussion

Under the condition of vertical incidence of a plane wave, when a one-dimensional coding pattern is employed (i.e., the coding varies only along a single direction, such as the x-axis or y-axis), the elevation angle can be simply expressed by Equation (1):

(1) $$\theta ={sin}^{-1}\left(\frac{\lambda }{\Gamma }\right)$$

where θ is the deflection angle, λ is the wavelength of the operating frequency in free space, and Γ is the period of the coding pattern. The elevation angle is directly related to the period length of the coding gradient sequence, and by adjusting the period length, reflection beams with different elevation angles can be achieved. Based on this equation, four coding arrays were designed with intervals of 10°, targeting angles of 40°, 30°, 20°, and 10°, respectively. The period lengths corresponding to each target angle were calculated according to Equation (1), and using the two designed phase gradient metasurface unit structures (with a phase difference of 180°) as the basic units, a 20 × 20 coding metasurface array was constructed. The four designed coding sequences are arranged one-dimensionally along the x-axis as follows: 00011100011100011100; 00001111000011110000; 00000011111100000011; 00000000000111111111. These coding sequences are extended along the y-axis to form coding arrays, denoted as N1, N2, N3, and N4, respectively. The coding periods calculated based on the target angles are 129 mm, 172 mm, 258 mm, and 473 mm, and the reflection angles calculated using Equation (1) are 41.6°, 29.9°, 19.4°, and 10.4°, with specific results shown in Table 2. This systematic approach demonstrates the precise control over beam deflection achievable through tailored coding sequences and period adjustments, highlighting the versatility of the proposed metasurface design.

**Figure 5:**
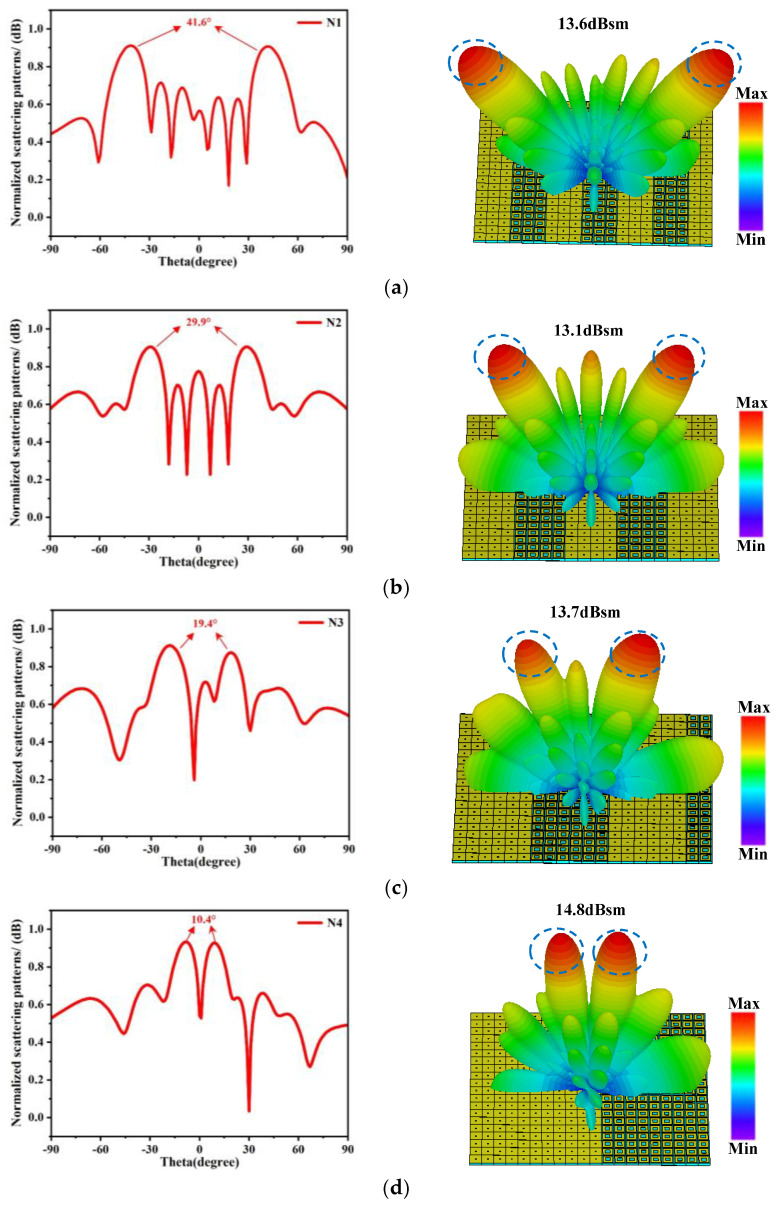
The simulation results of the four coding arrays, N1, N2, N3, and N4. (**a**) The radiation pattern and far-field radiation diagram of the N1. (**b**) The radiation pattern and far-field radiation diagram of the N2. (**c**) The radiation pattern and far-field radiation diagram of the N3. (**d**) The radiation pattern and far-field radiation diagram of the N4.

The simulation results for the N1, N2, N3, and N4 coding arrays are presented in Figure 5. As depicted in the figure, the incident plane wave generates symmetric dual beams upon interaction with the 1-bit coded metasurface. The symmetric reflected main beams for the four coding arrays are directed at angles of 41.6°, 29.9°, 19.4°, and 10.4°, respectively, aligning precisely with the calculated values in Table 2. The far-field radiation patterns illustrate the reflection intensities of the main lobes, with peak radiation intensities reaching 13.6 dBsm, 13.1 dBsm, 13.7 dBsm, and 14.8 dBsm for the four coding arrays, respectively. These results demonstrate the effective beam-splitting capability of the designed metasurface.

**Figure 6:**
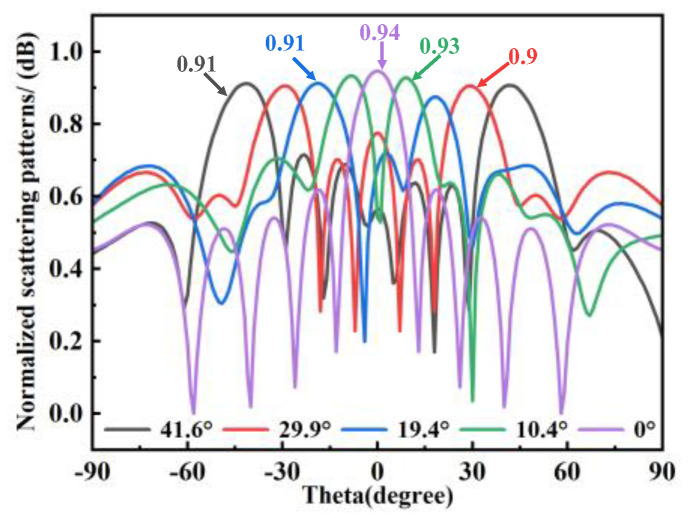
Normalized scattering patterns under different codings.

Figure 6 further highlights the performance of the four 1-bit coding arrays, which achieve symmetric dual beams at 10° intervals, specifically at 10.4°, 19.4°, 29.9°, and 41.6°. For comparison, the all-“0” coding state was simulated as a control case. In this configuration, all units are in the same state, resulting in no phase difference, and the reflection characteristics resemble those of a metallic plate, with the main beam reflected at 0°and a high reflection efficiency of 0.94 dB. In contrast, the N1, N2, N3, and N4 coding arrays, leveraging phase differences, exhibit a gradual reduction in the 0° normal reflection intensity (with a slight decrease of approximately 0.1 dB in reflection efficiency compared to the all-“0” case), while splitting into two symmetric beams about the xoz plane. As the energy shifts from the 0° main beam to the symmetric dual beams, the beam amplitude correspondingly decreases, confirming the successful implementation of beam steering through phase modulation. These findings underscore the versatility and precision of the proposed coding metasurface design in achieving controlled beam deflection, while maintaining high reflection efficiency.

To achieve more precise control over single-beam deflection angles, this study employs four phase gradient metasurface unit structures (with a phase difference of 90°) as the fundamental building blocks to construct a 20 × 20 2-bit coding metasurface. Five one-dimensional coding sequences, periodically arranged along the x-axis, were designed as follows: 00112233001122330011;00011122233300011122;00001111222233330000;00000111112222233333;00000011111122222233. These coding sequences were extended along the y-axis to form 20 × 20 coding arrays, designated as P1, P2, P3, P4, and P5, respectively. The 2-bit coding period Γ and the corresponding elevation angle θ were calculated using Equation (1), with detailed results summarized in Table 3.

**Figure 7:**
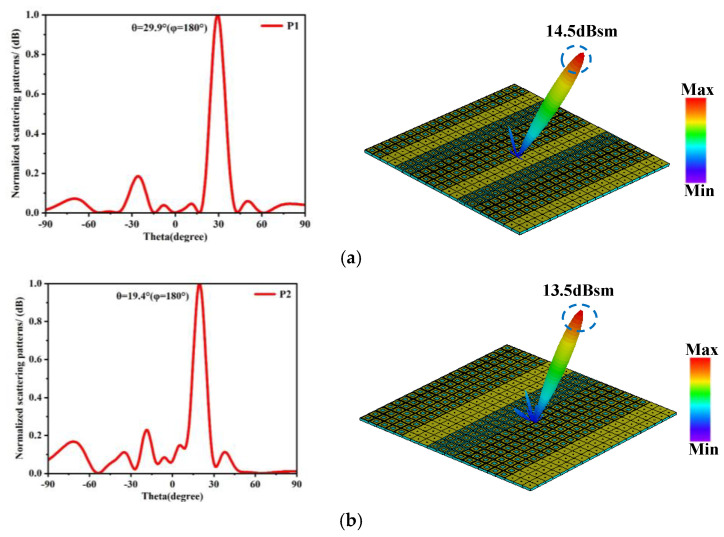

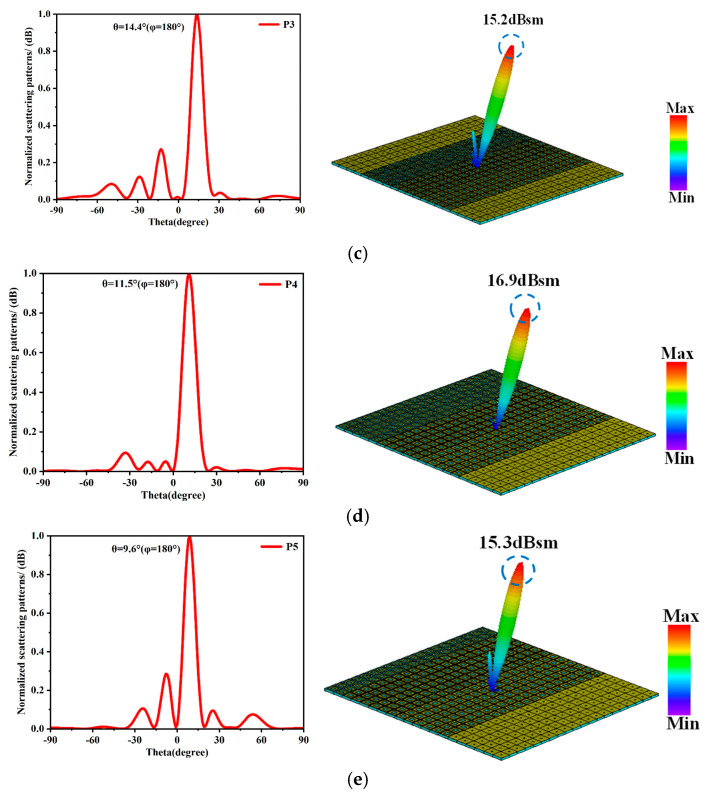
Far-field scattering patterns and radiation patterns obtained from simulations of the five coding metasurface. (**a**) Far-field scattering pattern and radiation pattern of the P1. (**b**) Far-field scattering pattern and radiation pattern of the P2. (**c**) Far-field scattering pattern and radiation pattern of the P3. (**d**) Far-field scattering pattern and radiation pattern of the P4. (**e**) Far-field scattering pattern and radiation pattern of the P5.

Simulation analysis was performed on the 2-bit coding arrays, and Figure 7 presents the far-field radiation patterns and Cartesian coordinate system radiation diagrams for arrays P1 to P5. The simulation results demonstrate that, under the 2-bit phase coding configuration, a vertically incident beam along the z-axis generates a single reflected beam. The main beams are directed at angles of 29.9°, 19.4°, 14.4°, 11.5°, and 9.6°, respectively, confirming the metasurface’s capability for precise single-beam deflection control. These findings highlight the enhanced flexibility and accuracy of the 2-bit coding approach in achieving tailored beam steering.

According to the principle described by Equation (1), the 2-bit periodic arrangement achieves the target elevation angle by specifying the coding period length. However, this method exhibits inherent limitations in terms of angular control precision and flexibility. To enhance the elevation angle control performance, this study employs Fourier convolution operations to process the basic coding sequences, enabling more versatile and precise control over the deflection angle of the reflected beam. The electric field distribution of the coding metasurface and its far-field radiation pattern are governed by a Fourier transform relationship, i.e., the coding array can be analogized to the time domain signal in digital signal processing, while the far-field radiation pattern corresponds to the frequency domain signal. According to the convolution theorem, the product of two signals in the time domain is equivalent to their convolution in the frequency domain, which can be expressed as [11]:

(2) $$f\left(t\right)\cdot g(t)\overset{FFT}{\to }f\left(\omega \right)*g(\omega )$$

where f(t) and g(t) represent the initial coding metasurface array and a phase gradient metasurface array, respectively. The convolution theorem implies that the product of the initial coding metasurface array and a phase gradient metasurface array will shift the reflected beam from its original direction. The elevation angle of the resulting coding metasurface can be expressed as:

(3) $$\theta ={sin}^{-1}\left(sin\theta_{1}\pm sin\theta_{2}\right)$$

where θ_1_ and θ_2_ are the elevation angles of the two initial coding metasurfaces. This approach provides a mathematical framework for achieving enhanced beam steering capabilities through the superposition of phase gradients, significantly improving the flexibility and precision of angle control in metasurface design.

**Figure 8:**
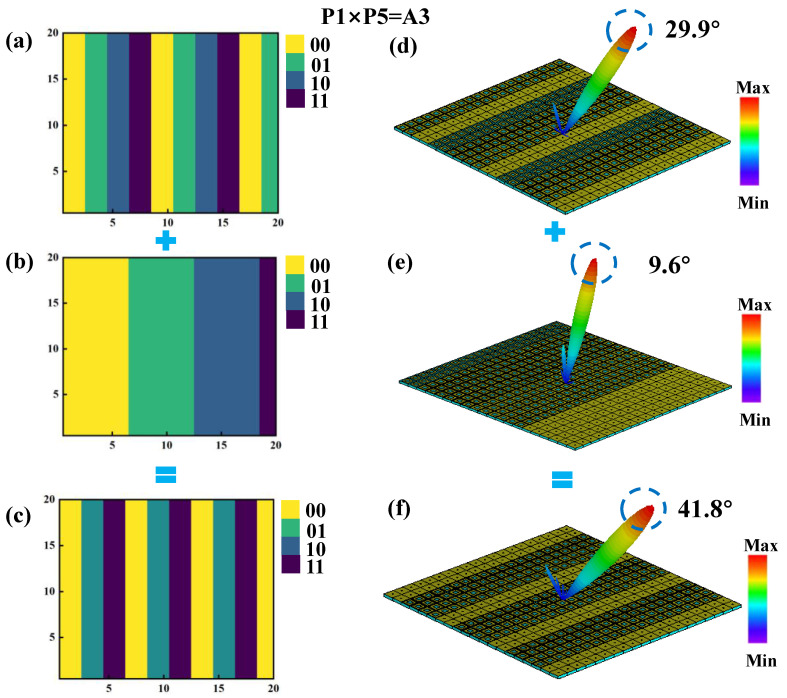
Schematic diagram of the convolution process of the coding metasurface under vertical incidence of a plane wave at 3.5 GHz. (**a**–**c**) Phase distribution diagrams of P1, P5, and A3. (**d**–**f**) Far-field radiation patterns.

By applying the convolution operation to the previously described P1 and P5 coding arrangements, the phase distributions of the P1 and P5 sequences are combined to form a new coding metasurface, designated as A3. According to Equation (3), under identical excitation conditions, the A3 metasurface generates a single reflected beam with a deflection angle of 41.8° and a main lobe reflection intensity of 15.8 dBsm, as illustrated in Figure 8f. This result demonstrates that, under one-dimensional phase gradient coding, the convolution of two coding arrangements with relatively limited beam control capabilities can produce a single-beam deflection with a significantly expanded angular range. This approach provides a practical and effective method for enhancing the control performance of reflected beams, offering greater flexibility in metasurface design for advanced beam steering applications.

Building on this methodology, the five phase gradient coding metasurfaces (P1, P2, P3, P4, and P5) were subjected to multiple superposition and hybrid addition–subtraction operations to derive additional arrangement configurations and controllable reflection angles, validated through computational simulations and experimental measurements. Linear superposition operations were applied to the selected metasurfaces, yielding the following sequences: A1 = P2 + P3 + P4, A2 = P1 + P3, A3 = P1 + P5, A4 = P1 + P2 − P4, A5 = P2 + P3, A6 = P3 + P5, and A7 = P1 − P4. The corresponding simulation and experimental results are summarized in Table 4. These seven distinct metasurface arrangements enable reflection angle variations spanning from 51.5° to 17.5°, with angular gradients controlled within 10° and main lobe reflection intensities ranging from 12 to 15.8 dBsm. The results demonstrate that convolution operations on the coding metasurface significantly expand the controllable range of reflection angles. While the traditional method of extending the period length achieves a maximum deflection angle of 41.6°, the application of convolution operations extends this range to 51.5°. Furthermore, Table 4 reveals that the main lobe amplitudes remain relatively stable after convolution operations, indicating that beam energy remains concentrated in the main beam direction. This ensures precise beam pointing accuracy, highlighting the effectiveness of the proposed approach in enhancing the performance and flexibility of metasurface-based beam steering systems.

Table 5 provides a comprehensive comparison of key performance metrics, including operating frequency, polarization, the maximum deflection angle, the experimental angle, half-power beamwidth, the number of beams, and bandwidth, across various studies. Compared to Ref. [20], both this paper and Ref. [20] have carried out measured verification at a deflection angle of 30°. The metasurface designed in this paper achieves an operating bandwidth of 214 MHz under a deflection of 30° and has dual-polarization characteristics. It can simultaneously support the independent manipulation of x-polarized and y-polarized incident waves, significantly improving its applicability and flexibility in multi-polarization scenarios. In contrast, Ref. [20] only supports single-polarization characteristics and has a narrow bandwidth, which limits its adaptability in practical applications.

In addition, compared to reference [17], although both operate in the 3.5 GHz frequency band, the metasurface designed in this paper can achieve a maximum deflection angle of up to 51.5° and supports precise single-beam manipulation. In the case of a 30° deflection, the half-power beamwidth of this paper is 6.3°, demonstrating excellent beam focusing ability. These performance advantages indicate that the metasurface designed in this paper has significant competitiveness in terms of deflection angle, bandwidth, polarization characteristics, and beam manipulation accuracy, providing an important reference for the design of future broadband and multi-polarized electromagnetic manipulation devices.

This work innovatively integrates multi-bit coding with a ring-shaped metasurface unit structure, achieving significant breakthroughs in engineering applications: a beam-steering system with precise 10° intervals (from 17.5° to 51.5°) has been successfully designed, meeting the practical requirements of 5G communication for beamforming. Compared to ref. [11], which only demonstrated basic scattering modes in the terahertz band, the innovations of this work include the following: (1) the adoption of multi-bit coding technology significantly enhances the flexibility and reliability of beam manipulation; (2) the ring-shaped metasurface unit structure markedly improves structural stability and fabrication feasibility; and (3) the design is optimized for the multi-polarization requirements and practical deployment scenarios of 5G communication, endowing the system with superior environmental adaptability. These technological advancements demonstrate the substantial advantages of the proposed approach in terms of engineering practicality and industrial potential.

## 4. Experimental Verification

Based on the proposed unit cell design, a coding array with a deflection angle of 30° was implemented using the coding sequence 00112233001122330011. Considering fabrication limitations, a prototype array comprising 20 × 20 unit cells with overall dimensions of 430 × 430 mm^2^ was fabricated for experimental validation.

**Figure 9:**
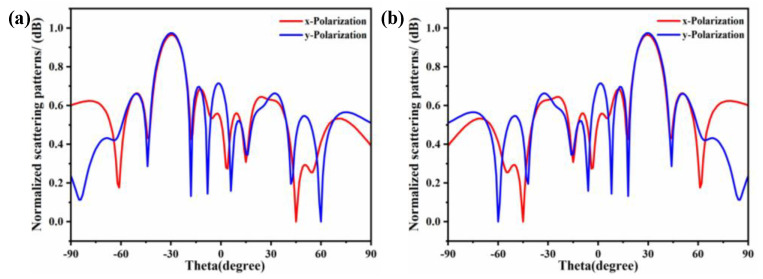
Radiation patterns under different polarization incidences. (**a**) Theta = −30°, Phi = 0°. (**b**) Theta = 30°, Phi = 180°.

The far-field radiation characteristics of the 2-bit coding array were numerically investigated under different polarization conditions, as illustrated in Figure 9. Specifically, Figure 9a presents the Cartesian radiation pattern of the metasurface under x-polarized incidence at (θ = −30°, φ = 0°), while Figure 9b demonstrates the corresponding pattern under y-polarized incidence at (θ = 30°, φ = 180°). The simulated results reveal nearly identical beam directions and radiation amplitudes for both polarization states, confirming the polarization-insensitive property of the proposed metasurface design. This characteristic is particularly advantageous for practical applications where polarization-independent performance is required.

**Figure 10:**
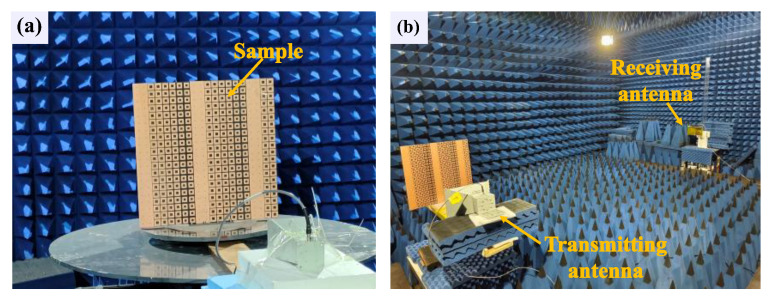
(**a**) Schematic diagram of the encoded metasurface sample. (**b**) Microwave anechoic chamber test environment.

To experimentally validate the beam-steering capability of the proposed coding metasurface in practical scenarios, a prototype comprising a 20 × 20 unit cell array with overall dimensions of 430 × 430 mm² was fabricated, as illustrated in Figure 10a,b. The far-field scattering characteristics were characterized in a microwave anechoic chamber using a custom-built measurement setup. The experimental configuration employed a linearly polarized horn antenna (operational frequency range: 1–18 GHz) as the transmitting source, with both the source and metasurface mounted on a computer-controlled rotational stage to enable 360° azimuthal scanning in the horizontal plane. A receiving antenna, identical in specifications to the transmitting antenna, was positioned at a far-field distance of approximately 5 m from the turntable to capture the scattered electromagnetic waves from the metasurface, as schematically depicted in Figure 10b. The beam-steering performance evaluation was conducted at 3.5 GHz using the free-space measurement technique, ensuring far-field conditions and minimizing environmental interference.

**Figure 11:**
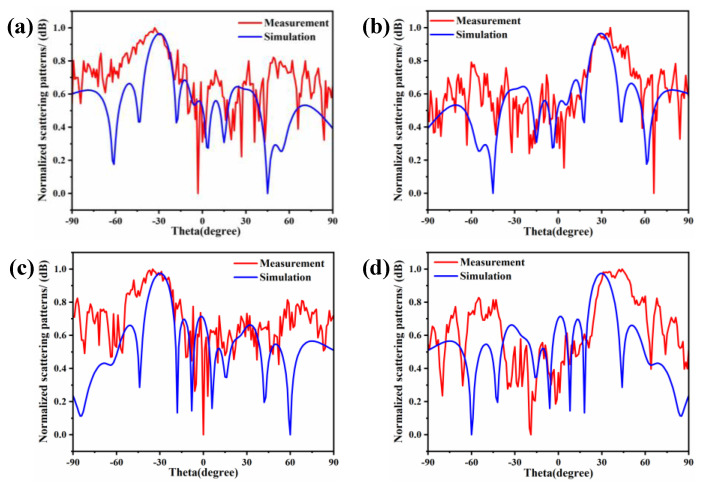
Comparison between measured and simulated results: (**a**,**b**) Far-field radiation patterns under x-polarized incident wave excitation with reflection angles of −30° (Phi = 0°) and 30° (Phi = 180°); (**c**,**d**) Far-field radiation patterns under y-polarized incident wave excitation with reflection angles of −30° (Phi = 0°) and 30° (Phi = 180°).

The far-field scattering characteristics of the fabricated coding metasurface array under different polarization excitations are systematically investigated in Figure 11. As demonstrated in Figure 11a, when illuminated by an x-polarized plane wave propagating along the +z direction at 3.5 GHz, the metasurface produces a well-defined scattered beam with a measured deflection angle of −30° in the azimuthal plane. By implementing appropriate phase gradient modifications through azimuthal angle φ adjustment, the beam direction is effectively steered to +30°, as shown in Figure 11b. Both simulated and experimental radiation patterns demonstrate excellent agreement with the theoretical predictions, confirming the successful implementation of directional beam control functionality. Figure 11c,d further reveals that, under y-polarized wave excitation with identical propagation direction and operating frequency, the metasurface maintains comparable beam-steering capability, achieving symmetric ±30° deflections through proper coding sequence optimization. A comprehensive comparison between the x-polarized and y-polarized excitation cases indicates remarkable polarization insensitivity of the designed structure, with measured radiation patterns showing negligible performance degradation (<1.5 dB sidelobe variation) between orthogonal polarization states. This polarization-independent beam manipulation capability confirms the dual-polarized operation characteristics of the proposed coding metasurface architecture, while maintaining stable beam deflection accuracy within ±1.2° across all tested polarization conditions.

**Figure 12:**
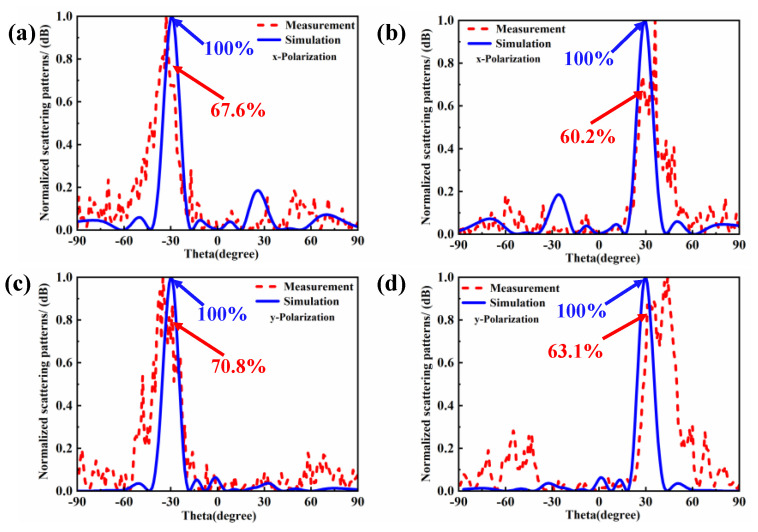
The simulated and measured power patterns in linear scale at 3.5 GHz. (**a**,**b**) X-polarized incident wave with reflection angles of ±30°. (**c**,**d**) Y-polarized incident wave with reflection angles of ±30°.

In order to compare the deflection efficiency under simulation and measurement conditions, we used the normalized pattern in linear values for evaluation. Figure 12 shows the power patterns under simulation and measurement conditions. Figure 12a and Figure 12b, respectively, show the simulated and measured beam deflection efficiency at a frequency of 3.5 GHz for an x-polarized incident wave. Under the coding array with an angle range of ±30 degrees, the measured deflection efficiencies are 67.6% and 60.2%, respectively. Figure 12c and Figure 12d show the results for a y-polarized incident wave, and the measured deflection efficiencies are 70.8% and 63.1%, respectively. As illustrated in Figure 12, for both x-polarization and y-polarization, the measured deflection efficiency is lower than the simulation result. This difference may be attributed to factors such as losses in the actual environment, manufacturing errors, or measurement errors. Within the deflection range of ±30°, the deflection efficiency ranges from 60% to 70%, indicating that the coding array can still maintain a relatively high deflection performance at larger angles.

Table 6 compares phased array antennas [24,25], conventional metasurface beamformers [26,27], and the metasurface in this work across several key performance metrics. Regarding hardware complexity, the proposed design employs a compact unit structure composed of only three layers, significantly reducing complexity compared to the sophisticated architecture of phased array antennas [24,25]. Regarding computing complexity, the proposed system is undemanding, contrasting with the intensive computational requirements of conventional metasurface beamformers [26,27]. The power consumption of our system is particularly noteworthy. While phased array antennas demand numerous phase shifters, resulting in high power consumption [24,25], our design utilizes a passive structure, thereby achieving dramatically reduced power requirements. Furthermore, manufacturing costs are maintained at inexpensive levels versus the costly implementation of phased arrays [24,25]. Finally, the angular coverage range is extended to ±51.5°, surpassing the ±30° capability of conventional metasurface beamformers [26,27]. These advantages collectively demonstrate the superior performance and practicality of our ring-shaped metasurface for 5G communication systems.

## 5. Conclusions

In this work, we have designed and demonstrated an annular unit element coding metasurface operating at 3.5 GHz for advanced beam manipulation in wireless communication systems. The proposed metasurface enables flexible control of electromagnetic waves, supporting both single-beam and dual-beam configurations through optimized encoding sequences. By employing a convolution-based encoding method, multiple phase gradients were realized, achieving anomalous reflection of vertically incident plane waves with seven distinct deflection angles ranging from 51.5° to 17.5°. The metasurface design allows precise beam focusing in targeted areas, significantly enhancing signal reception sensitivity and transmission efficiency. Experimental results validate the design’s capability to improve 5G wireless communication channel performance and address signal blind spots in localized regions. This work provides a promising approach for developing reconfigurable intelligent surfaces in next-generation communication systems, with potential applications in beamforming, coverage extension, and multi-user spatial multiplexing. Future research will focus on expanding the operational bandwidth and integrating active components for real-time beam control.

## Figures and Tables

**Table 1:** Schematic diagrams of 1-bit and 2-bit coding unit cells.

a/(mm)	w/(mm)	phase/(°)	1-bit Phase Coding	2-bit Phase Coding
21.5	9.68	0	0	00
18.8	4.85	90	---	01
17.5	4.46	180	1	10
15.3	3.9	271	---	11

**Table 2:** Coding sequences for specific angles.

Sequence Name	Coding Sequence	Γ (mm)	θ (°)
N1	00011100011100011100	129	41.6
N2	00001111000011110000	172	29.9
N3	00000011111100000011	258	19.4
N4	00000000000111111111	473	10.4

**Table 3:** Period Γ and elevation angle θ of different 2-bit coding sequences.

Sequence Name	Coding Sequence	Γ (mm)	θ (°)
P1	00112233001122330011	172	29.9
P2	00011122233300011122	258	19.4
P3	00001111222233330000	344	14.4
P4	00000111112222233333	430	11.5
P5	00000011111122222233	516	9.6

**Table 4:** Reflection angles and main lobe reflection intensities under different arrangement methods.

	A × B = C	One-Dimensional Radiation Pattern	Far-Field Radiation Pattern
(a)	P2 + P3 + P4 = A1	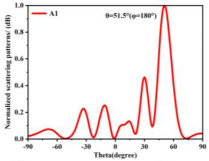	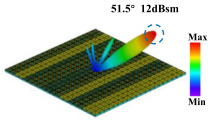
(b)	P1 + P3 = A2	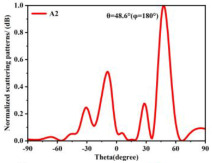	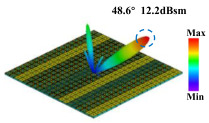
(c)	P1 + P5 = A3	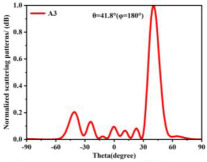	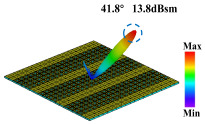
(d)	P1 + P2 − P4 = A4	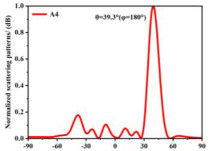	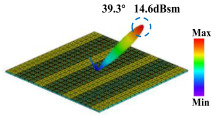
(e)	P2 + P3 = A5	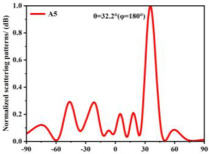	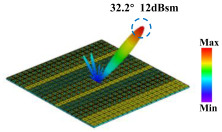
(f)	P3 + P5 = A6	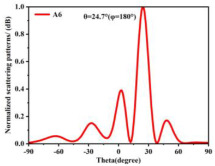	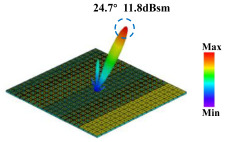
(g)	P1 − P4 = A7	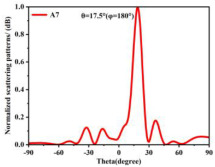	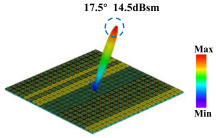

**Table 5:** Comparison between this work and previous studies.

Ref.	[11]	[17]	[21]	[22]	[23]	[20]	This Work
Frequency	1 THz	3.5 GHz	15 GHz	10 GHz	7.17 GHz	28 GHz	3.5 GHz
Polarization	Single	Single	Dual	Single	Single	Single	Dual
Maximum θ (°)	63.2°	30°	18.2°	48.59°	28°	45°	51.5°
Experimental θ (°)	41.5°	30°	18.2°	22.2°	28°	30°	30°
Half-power beam width	---	---	2°	---	7°	10.7°	6.3°
Number of beams	1	2	1	4	1	1	1
Bandwidth	0.01 THz	300 MHz	40 MHz	400 MHz	100 MHz	50 MHz	214 MHz

**Table 6:** Comparison of key indicators among this work, phased array antennas, and conventional metasurface beamformers.

Ref.	Hardware Complexity	Computing Complexity	Power Consumption	Manufacturing Cost	Angular Coverage Range
[24]	sophisticated	intensive	high	costly	±45°
[25]	sophisticated	intensive	high	costly	±38°
[26]	sophisticated	intensive	low	inexpensive	22.5°
[27]	sophisticated	intensive	low	inexpensive	±30°
This work	uncomplicated	undemanding	low	inexpensive	±51.5°

## Data Availability

The data presented in this study are available on request from the corresponding author.
